# Elucidating ‘Transfer‐Lithiation’ from Graphite to Si within Composite Anodes during Pre‐Lithiation and Regular Charging

**DOI:** 10.1002/cssc.202401290

**Published:** 2024-12-19

**Authors:** Lars Frankenstein, Pascal Jan Glomb, Marvin Mohrhardt, Steffen Böckmann, Leon Focks, Aurora Gomez‐Martin, Tobias Placke, Michael Ryan Hansen, Martin Winter, Johannes Kasnatscheew

**Affiliations:** ^1^ MEET Battery Research Center Institute of Physical Chemistry University of Münster Corrensstraße 46 48149 Münster Germany; ^2^ Helmholtz Institute Münster IMD-4 Forschungszentrum Jülich GmbH Corrensstraße 46 48149 Münster Germany; ^3^ Institute of Physical Chemistry University of Münster Corrensstraße 28/30 48149 Münster Germany

**Keywords:** Si-based anodes, Pre-lithiation, Local element, Si overlithiation, Li plating

## Abstract

Si‐based anodes can increase specific energy and energy density of Li ion batteries. However, the volume‐induced material stress and capacity loss necessitates only a partial Si utilization within composite anodes, typically with state‐of‐the‐art graphite, so called Si/Gr composites. In this work, various Si nanowires (SiNWs), a promising Si architecture for these composites, are investigated and modified *via* pre‐lithiation. Though, charged pre‐lithiated anodes show potentials below 0 V *vs*. Li|Li^+^ in the initial cycles, they do not show indications for metallic Li, which is likely a hint for a triggered surface Li depletion in course of a continuous “transfer‐lithiation” from lithiated Gr to Si, which is indicated by decreasing LiC_6_ and increasing Li_*x*_Si_*y*_ signals *via* nuclear magnetic resonance (NMR), X‐ray diffraction (XRD) as well as shifts in capacities of respective voltage plateaus during discharge after storage. A relevant contribution of self‐discharge is unlikely as shown by a stable open‐circuit‐voltage during storage in charged state and similar subsequent discharge capacities, being consequently a hint for an *intra‐*electrode capacity shift. The process of transfer lithiation is finally validated *via* solid‐state ^7^Li NMR for varied Si morphology, *i. e*., amorphous and crystalline, as well as during pre‐lithiation with passivated lithium metal powder (PLMP).

## Graphical abstract

The TOC illustrates the benefit of Si‐addition to graphite‐based anodes in terms of energy density. However, even in small ratios the Si can get overlithiated and degrade in course of transfer lithiation, *i. e*., from graphite to Si, as observed *via* nuclear magnetic resonance spectroscopy (NMR) or respective capacity shifts of Si and graphite in charged state.

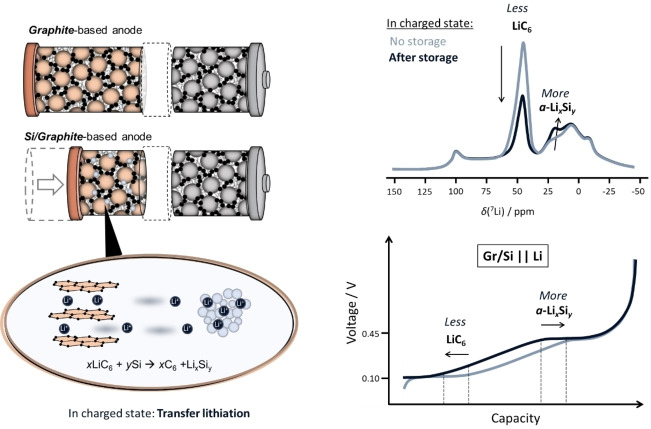

## Introduction

State‐of‐the‐art (SOTA) Li ion batteries (LIBs) with graphite (Gr)‐based negative electrodes (NEs) require an increase in energy density for a more successful market penetration of electric vehicles (EVs).[^1^, ^2^, ^3^] Current R&D focuses, among other, on alternative NE materials such as metal oxides and lithium storage‐metals and ‐alloys (*e. g*., Mg, Al, Sn,),[^4^, ^5^, ^6^] in particular on silicon (Si) due to relatively high abundancy and higher specific/volumetric capacities (3579 mAh g^−1^, 2194 mAh cm^−3^) compared to Gr (372 mAh g^−1^, 719 mAh cm^−3^).[^4^, ^5^, ^7^, ^8^]

However, Si suffers from a volume expansion of up to 280 % during full (de–)lithiation^[9]^ leading to cracks, detachment from the current collector, increase in specific surface area (SSA), finally active lithium losses (ALL) in course of continuous electrolyte decomposition and reformation/repair of the passivation layer, *i. e*., the so‐called solid electrolyte interphase (SEI^[10]^), which decreases cell capacity and cycle life.[^9^, ^11^] Typical approaches are either the combination of Si with SOTA Gr as an Si/Gr composite, and/or downsizing Si active material towards, *e. g*., silicon nanoparticles (SiNPs) or silicon nanowires (SiNWs) for reasons of mechanical stability and buffering material stress.[^12^, ^13^, ^14^, ^15^, ^16^, ^17^, ^18^] While many material aspects are reported like critical diameter of cracking and/or splitting‐into‐parts, *i. e*., 150 nm for SiNPs,^[19]^ while 300 nm^[20]^ and 216 nm^[21]^ for SiNWs, the SEI aspects,^[22]^ or individual behavior of Si and Gr within composite,^[23]^ as well as their interactions remain rather unclear.[^24^, ^25^]

Deposited Li on NE, *e. g*., after charge at low‐temperature, is known to re‐insert/re‐intercalate in Si/Gr NEs, subsequently to relocate from Gr to Si during relaxation,[^26^, ^27^, ^28^, ^29^, ^30^] and enhance the NE potential.^[23]^ Different working potentials between Gr and Si, thus thermodynamics (*Gibbs* energy) can be suggested to be the driving force.^[23]^ However, interactions between Gr and Si during charge/discharge cycling are only reported in full cells, *i. e*., where active Li amount is limited,^[31]^ or on model‐like blended electrodes, *i. e*., parallelly connected individual Gr and *a*‐Si thin film electrodes.^[23]^

Herein, this reaction between Gr and Si as a local element, which we name ‘transfer lithiation’ phenomenon is systematically investigated in composites of SiNW/Gr‐based electrodes with a particular focus on storage impact and the Si material itself, *e. g*., the SiNW diameter as well as Si morphology (*c*‐Si *vs. a*‐Si).

## Experimental

### Electrode Preparation

Electrodes with a composition of 85 wt.% of SiNW/Gr (22 % (A), 17 % (B) and 19 % (C) Si content, based on practical specific de‐lithiation capacity; SMG A5 *Hitachi Ltd. Corporation*) active material with variation in diameter size provided by *Enwires SAS*, 5 wt % of conductive agent (Super C65, *Imerys Graphite & Carbon*), 7.7 wt % of sodium‐carboxymethyl cellulose (Na‐CMC, *Walocel CRT 2000 PPA 12, Dow Wolff Cellulosics*) and 2.3 wt % of neutralized lithium polyacrylic acid (LiPAA, *Sigma Aldrich*) as binder materials were prepared on smooth copper foil as current collector. Due to their unique synthesis, there are some minor fluctuations in SiNW growth/deposition leading to slight variations in Si content as well as corresponding capacity. First, LiPAA was prepared by dissolving PAA and lithium hydroxide in deionized water. Na‐CMC was subsequently added and the solution was dispersed for 5 min at 1700 rpm using a *Thinky* mixer. Afterwards, Super C65 was added and the binder solution was further dispersed at 1700 rpm to create a black paste for 5 minutes. Subsequently, active materials (SiNW/Gr‐A, B and C; described in Table 1) were added and dispersed for 10 min at 1700 rpm. The resulting electrode paste was coated on smooth copper foil using a doctor blade technique. A blade gap of 70–80 μm was chosen so that the electrodes exhibited an active mass loading of ~2.0 mg cm^−2^ (1.5 mAh cm^−2^). Electrode sheets were pre‐dried at 70 °C in ambient air for 60 minutes and electrode discs (Ø=12 and 15 mm) were punched which were further dried at 120 °C for 12 hours under reduced pressure. After drying, the electrodes were weighed and stored in a dry room (≤0.02 % moisture).

**Table 1:** Relevant data of the active materials and 1^st^ cycle electrochemical properties of SiNW/Gr active materials investigated in half‐cells.

Sample	SiNW/Gr‐A	SiNW/Gr‐B	SiNW/Gr‐C
Thickness/nm	18	66	59
SSA/m^2^ g^−1^	34.4	15.5	60.2
IC_*Eff*_/%	78.95±0.11	82.44±1.17	75.69±1.37
Specific de‐lithiation capacity/mAh g^−1^	809.3±2.5	702.4±8.6	755.9±4.4
Capacity Retention after cycling/%	93	91	85

LiNi_0.6_Co_0.2_Mn_0.2_O_2_ (NCM622, *Shanshan Tech Co*.) positive electrodes were prepared using 95 wt % AM, 2 wt % conductive agent (Super C65) and 3 wt % polyvinylidene difluoride (PVdF) as binder material. Firstly, PVdF was dissolved in *N*‐methyl‐2‐pyrrolidinone (NMP) using a Dispermat LC30 at a speed of 1500 rpm. C65 conductive agent and NCM622 were subsequently added and the mixture was stirred for 60 minutes at 10,000 rpm. The so‐prepared electrode paste was coated on aluminium foil using a doctor blade technique. The electrodes exhibited an active mass loading of ~8.9 mg cm^−2^ (1.5 mAh cm^−2^). Electrode sheets were pre‐dried at 70 °C in ambient air for 60 minutes, calendared to reach a porosity of 35 % and electrode discs (Ø=14 mm) were punched which were further dried at 120 °C for 12 hours under reduced pressure. After drying, the electrodes were weighed and stored in a dry room (≤0.02 % moisture).

### Cell Assembly

Electrochemical characterization of the active materials was performed in a three‐electrode setup using T‐shaped *Swagelok* cells. SiNW/Gr materials were used as working electrode (WE, Ø=12 mm) while lithium metal discs were used as counter and reference electrode (CE and RE, Ø=12 mm). The interior of the cell was insulated by *Mylar* foil to guarantee electronic isolation of the electrodes. WE and CE were isolated by three layered *Freudenberg FS2190* polyolefin (Ø=13 mm) separator which was soaked with 120 μL electrolyte of 1 M lithium hexafluorophosphate (LiPF_6_) in a mixture of ethylene carbonate (EC) and ethyl methyl carbonate (EMC) in a ratio of 3 : 7 by weight (LP57) with 10 wt % of fluoroethylene carbonate (FEC) as additive. RE was separated from WE and CE by 3 layered *FS2190* (Ø=8 mm) which was soaked with 60 μL of the electrolyte.

Long‐term electrochemical experiments of NMC622 || SiNW/Gr were performed in *CR2032*‐type coin cells in two‐electrode configuration. SiNW/Gr electrodes (Ø=15 mm) were used as negative electrode while NMC622 electrodes were used as positive electrode (Ø=14 mm). The negative to positive electrode (N : P) ratio was set to 1.0 and held constant during all experiments. Three layered *FS2190* separator (Ø=16 mm) was used and soaked with 150 μL LP57+10 wt % FEC. All positive electrodes had a respective areal capacity of 1.5 mAh cm^−2^.

### Galvanostatic Charge/Discharge Cycling

Electrochemical investigations were carried out on a *Maccor Series 4000 automated test system* (*Maccor Inc*.) using three cells for each experiment. To determine the mechanical stability of various SiNW diameter sizes, SiNW/Gr || Li metal three electrode cells were used for long‐term cycling. The cut‐off potentials were set to 0.01 V and 1.5 V *vs*. Li|Li^+^. Two cycles at 0.04 C (28 mA g^−1^) were applied followed by 50 cycles at 0.33 C (231 mA g^−1^) with a constant potential (CP) step at 0.01 V until the current dropped below 0.1 C (70 mA g^−1^). The changes in potential hysteresis were used to determine the stability of each material.

Electrochemical pre‐lithiation of SiNW/Gr‐A was performed to reach degrees of pre‐lithiation (DOPL) ranging from 0 % (pristine) to 50 % based on the initial specific delithiation capacity of ~800 mAh g^−1^. Therefore, the 1^st^ cycle irreversible capacity loss of ~216 mAh g^−1^ was considered. Details about the pre‐lithiation are given in results and discussion section. The pre‐lithiation was adjusted in CR2032‐type coin cells and based on the first cycle specific de‐lithiation capacity (*q_D_*). SiNW/Gr‐A had a first cycle specific lithiation capacity of ~916 mAh g^−1^ and a first cycle de‐lithiation capacity of ~776 mAh g^−1^. Therefore, an initial loss of 140 mAh g^−1^ (≙ irreversible capacity (*q_Irr_*); 18 % of the practical capacity of 776 mAh g^−1^) had to be included in the calculation. DOPLs from 10 % to 50 % were achieved by lithiation up to (*q_DOPL_*) 218 mAh g^−1^ (10 %), 295 mAh g^−1^ (20 %), 373 mAh g^−1^ (30 %), 450 mAh g^−1^ (40 %) and 528 mAh g^−1^ (50 %), respectively.

Furthermore, the electrochemical performance of each material was investigated in NMC622 || SiNW/Gr full cells in two electrode setup. Two cycles at 0.04 C (6.8 mA g^−1^) were applied followed by 50 cycles at 0.33 C (56.1 mA g^−1^) with a constant voltage (CV) step at 0.01 V until the current dropped below 0.1 C (17 mA g^−1^).

### Scanning Electron Microscopy and Energy‐dispersive X‐ray Spectroscopy

To investigate structural changes of the electrodes during cycling compared to the pristine electrodes, scanning electron microscopy (SEM) measurements were performed on a *Carl Zeiss AURIGA* Scanning electron microscope (*Carl Zeiss Microscopy GmbH*) with an acceleration voltage of 3 kV. The cycled electrodes were washed three times by 100 μL dimethyl carbonate (DMC) to remove residual salts and transferred in a vacuum sealed vessel to the SEM device. To investigate the elemental composition of the electrodes surface, energy dispersive X‐ray (EDX) measurements were performed at an acceleration voltage of 10 kV using an EDX detector (*Oxford Instruments*).

### Analysis via X‐ray Diffraction

*In situ* powder X‐ray diffraction (XRD) measurements were performed with fully lithiated SiNW/Gr electrodes on a *Bruker D8 Advance* X‐ray diffractometer with Ni‐filtered Cu‐Kα wavelength of 0.154 nm and a divergence slit of 0.5° in an *in situ* cell with 10 mm inspection area (*ECO Opto Std; EL‐Cell*). The lithiated active material of the electrodes faced a beryllium (Be) disk (thickness=250 μm; *Brush Wellmann GmbH*). The patterns were measured in a *2θ* range from 10–40° with a step size of 0.04° and a step time of 1.8 s. A total number of 737 steps (22.1 min per scan) was conducted for the whole measurement and a rest step of 3.9 min was used to limit one scan to 25 min. A reference measurement ranging from 10–60° was conducted to adjust the measured patterns with regards to the reflex of Be (*101*).^[32]^

### Analysis via Solid State ^7^Li NMR

Magic angle spinning (MAS) ^7^Li nuclear magnetic resonance (NMR) spectroscopy experiments were performed at room temperature with an *AVANCE III* 200 MHz spectrometer operating at a magnetic field of 4.7 T leading to a Larmor frequency of 77.9 MHz for ^7^Li nucleus. All spectra were recorded with a relaxation delay of 1 s and 2000 scans. The NMR spectra were referenced to a 1 M lithium chloride (LiCl) solution and its isotropic chemical shift was set δ_iso_=0 ppm. Obtained data were analyzed by *BRUKER Topspin 3.5* software for applying automatic phase correction.

All static solid‐state ^7^Li NMR spectra were acquired on a Bruker Avance Neo spectrometer operating with a Bruker wide bore magnet with a static field of 11.74 T (^7^Li resonance frequency of 194.47 MHz) and a commercial Bruker 2.5 mm H/F/X MAS DVT probe under static conditions. Samples were packed in 2.5 mm o. d. ZrO_2_ rotors. The ^7^Li chemical shift was referenced against LiCl solution (aq., 1 mol L^−1^) to 0 ppm by the replacement method. Pulse powers were calibrated directly one the sample to give a 90° pulse of 6 μs. To observe transfer kinetics, the temperature was set to 328 K (calibrated with pure MeOH beforehand [cite: 10.1021/ac50158a064 and 10.1016/s0066‐4103(02)45009‐0]) and one pulse spectra were acquired every 200 s or 300 s (8 scans, 16 s recycle delay). No change in the spectra was observed at ambient temperature due to hindered diffusion pathways.

## Results and Discussion

### Characterization of Different Silicon Nanowire/Graphite Materials

Silicon nanowires (SiNWs) are obtained *via* synthetic growth on graphite (Gr) as reported in literature.[^33^, ^34^] SiNW/Gr‐A & B, obtained by gold nanoparticles (Au‐NPs), differ in SiNW thickness and specific surface area (SSA) as shown in Table 1. In contrast, the carbon coated SiNW/Gr‐C, which is obtained *via* a more cost‐efficient tin chloride (SnCl_2_) as activator (“catalyst”), exhibits a similar thickness as SiNW/Gr‐B, but a higher SSA and is investigated for reasons of validation, as well.

**Figure 1:**
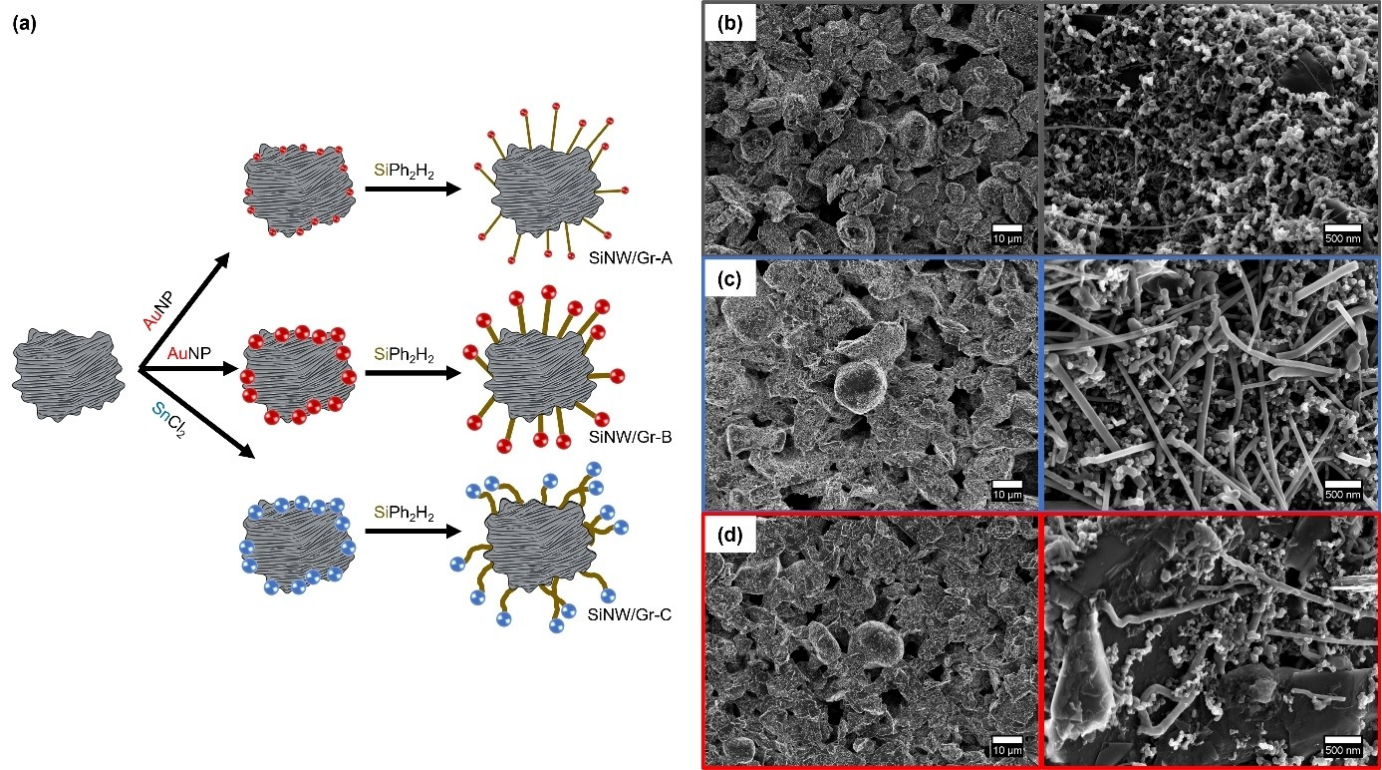
**a)** Schematic illustration of the synthesis of the different SiNW/Gr composites. SEM figures with varied magnifications for **b)** SiNW/Gr‐A, **c)** SiNW/Gr‐B and **d)** SiNW/Gr‐C. Materials obtained by Au‐catalyst have a straighter (rectilinear) morphology and the increased SiNW diameter size is clearly evident for SiNW/Gr‐B/C, while SiNW/Gr ‐A reveals thin filaments. The morphology of Sn‐derived SiNWs differs, *i. e*., is more curved and angular.

SiNW/Gr‐A and B indicate a more homogeneous distribution of Si on top of the graphite compared to SiNW/Gr‐C, as seen in Figure 1. The SiNWs in samples A (thin filaments) and B are observed to have a straighter morphology while NWs of the sample C are more angular and curved. Reflection data *via* powder X‐ray diffraction (XRD) (Figure S1) indicate crystalline Si (*c*‐Si) for all samples. Additional reflections for SiNW/Gr‐C at 30.5°, 32.0° and 43.8° can be attributed to *200*, *101* and *220* reflections of Sn catalyst (Figure 1a). Sn presence is (0.1 at %±0.1 at %) is also indicated *via* EDX analysis (Figure S2).

### Electrochemical Evaluation of SiNW‐based NEs in Li Half‐cells

**Figure 2:**
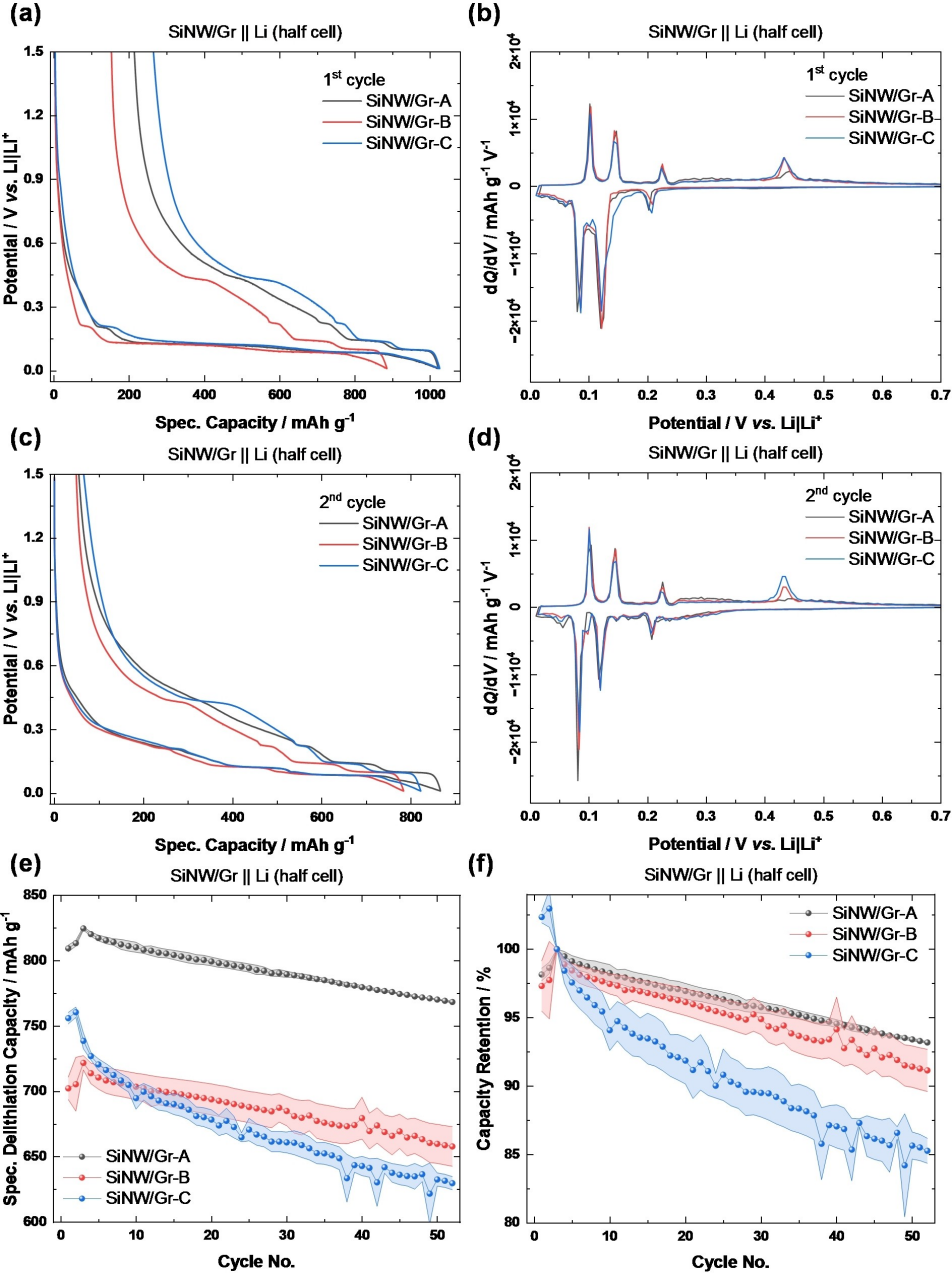
Charge/discharge potential *vs*. capacity profiles of **a)** first and **c)** second cycle (40 mA g^−1^; 0.01–1.5 V) for varied SiNWs in SiNW/Gr||Li (−half‐cell) with respective dQ/dV *vs*. voltage plots in **b)** and **d)**. **e)** Charge/discharge cycling, conducted with 231 mA g^−1^ (≈0.33 C) after formation. **f**) Corresponding capacity retention.

Charge/discharge potential profiles of the first and second cycle in a SiNW/Gr || Li half cell are shown in Figure 2 **a** and **c**, respectively. The potential plateaus indicate characteristic (de–)lithiation of Gr and Si, which are also plotted *via* differential capacity (d*Q*/d*V*)vs. potential plots in Figure 2 **b** and **d** for reasons of clarity. During lithiation, the plateaus/peaks can be linked with formation of Li_*x*_C_*n*_ phases (intercalation stages) at 202 mV, 149 mV, 105 mV and 84 mV, being similar to literature (200 mV, 140 mV, 110 mV and 80 mV).^[35]^ The plateau/peak at 120 mV as the result of transformation of *c‐*Si to amorphous Si (*a*‐Li_*x*_Si_*y*_) overlaps with aforementioned Gr lithiation at 110 mV. With further lithiation below 50 mV, *a*–Li_*x*_Si_*y*_ recrystallizes to *c*–Li_15_Si_4_ and is accompanied with severe volume expansion up to ~280 %.^[8]^ During de–lithiation, three plateaus/peaks for Gr de–intercalation are observed at 102 mV, 147 mV and 224 mV and one for de–lithiation of *c*‐Li_15_Si_4_ to *a*‐Si at 450 mV while the latter peak is less concise for SiNW/Gr‐A, likely as a result of small diameter size of SiNWs (18 nm) and higher amorphization.

The pattern for Si (de−)lithiation process is different for second cycle (Figure 2c and d). A new (broader) peak during lithiation emerge at ~250 mV and 100 mV indicating *a*–Si+*x*Li to *a*‐Li_*x*_Si as well as *a*‐Li_*x*_Si+*y*Li to *a*–Li_*x*+*y*_Si, both single–phase transformations. It is still recrystallizing below 50 mV to from *c*‐Li_*15*_Si_*4*_. Despite the single–phase formation of *a*–Li_*x*_Si and *a*–Li_*x*+*y*_Si, the only peak observed during de–lithiation is again at 450 mV, representing the two–phase transformation of *c*–Li_15_Si_4_ to *a*–Si.^[9]^ Overall, the peaks for (de–)lithiation of Si are broader than for graphite (de−)intercalation due to kinetically more hampered ‘sloping plateaus’ (Figure 2).

The capacity fade (Figure 2e and f) in Li‐half‐cells (= includes Li reservoir from excess Li metal)[^11^, ^36^, ^37^] can be attributed to structural aspects, while the initial Coulombic efficiency (IC_*Eff*_) predominantly results from electrolyte reduction and on SSA (Table 1).^[38]^ Though the higher first cycle specific de‐lithiation capacity of 809 mAh g^−1^ can be attributed to a slightly higher Si content in SiNW/Gr‐A (see experimental section) in the course of the synthesis procedure (Figure 1), the highest capacity retention (93 %) is observed after 50 cycles and can be related with thin diameter of the SiNWs, thus lower material stress in course of volume expansion.^[21]^

### Evaluation of Silicon Nanowire‐based NEs in Full‐cells

**Figure 3:**
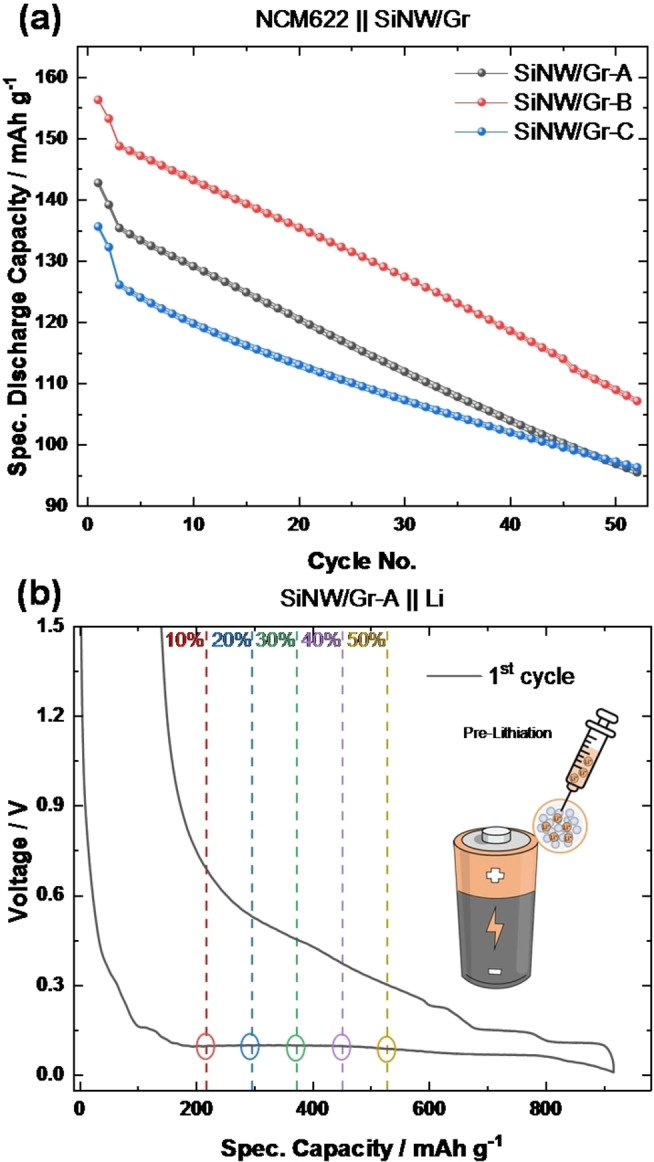
**a)** Specific discharge capacities of NCM622 || SiNW/Gr full‐cells as a function of cycle number and **b)** illustration of the effect of various degrees of electrochemical pre‐lithiation of SiNW/Gr‐A in reference to the specific discharge capacity at 0.1 C (80 mA g^−1^) with colored markers to demonstrate the pre‐lithiation to different DOPLs (218 mAh g^−1^ (10 %, red), 295 mAh g^−1^ (20 %, blue), 373 mAh g^−1^ (30 %, green), 450 mAh g^−1^ (40 %, purple) and 528 mAh g^−1^ (50 %, yellow), respectively).

Figure 3a depicts NCM622 || SiNW/Gr full‐cells, where the impact of SSA and losses in active lithium is obvious and show lowest specific capacities for SiNW/Gr‐C‐based NEs and highest for SiNW/Gr‐B (following SSA and IC_*Eff*._ according to Table 1).

Addition of an extra Li reservoir prior to cycling *via* a so‐called pre‐lithiation process can counteract the lithium losses and improve cycle life. SiNW/Gr‐A, being favorable from material point of view is pre‐lithiated electrochemically to different degrees of pre‐lithiation (DOPL). The DOPLs range from 0 % (pristine) to 50 % (Figure 3b), *i. e*., up to 388 mAh g^−1^, defined and calculated from 1^st^ cycle specific discharge capacity; to achieve the “net” reversible DOPLs during discharge, the gross pre‐lithiation is intentionally higher to compensate for irreversible Li losses, as defined in Figure 3.

### Electrochemical Pre‐lithiation of SiNW/Gr‐A

**Figure 4:**
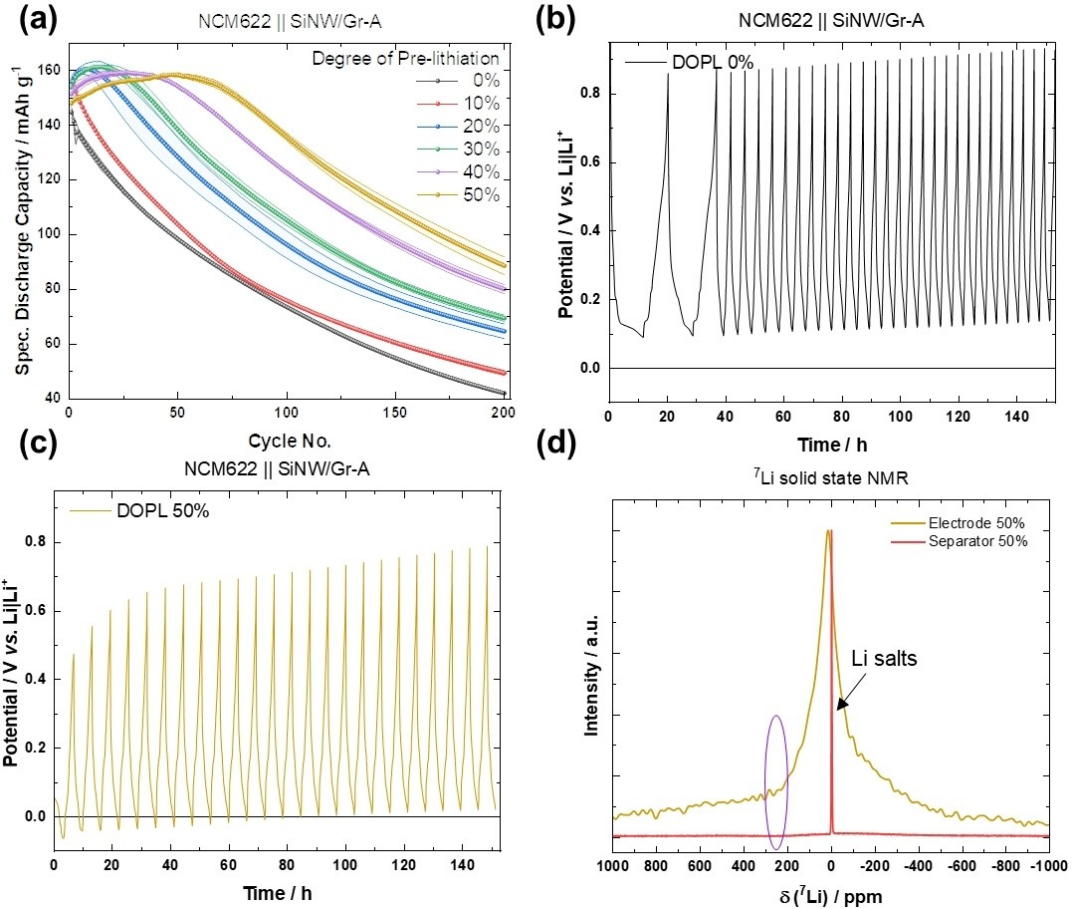
**a)** Specific discharge capacities over cycling of NCM622 || SiNW/Gr‐A full‐cells with different DOPLs. Evolution of NE potential in three‐electrode NCM622 || SiNW/Gr‐A full‐cells at DOPL of **b)** 0 % and **c)** 50 % and **d)** *ex situ* NMR measurements of pre‐lithiated NE and separator after cycling indicating no Li metal (purple circle at 250 ppm^[39]^).

Figure 4a displays the obtained specific discharge capacities of pre‐lithiated SiNW/Gr‐A in full‐cell configuration showing prolonged cycle life with higher DOPL. Interestingly, the initial specific discharge capacity is even lower for highest DOPL (148 mAh g^−1^ at 50 % DOPL) compared to lower DOPL (156 mAh g^−1^ at 10 % DOPL) and can be correlated with NE over‐lithiation. In pristine NCM622 || SiNW/Gr‐A full‐cells with an IC_*Eff*_ of only 73 %, Li addition above 27 % (DOPL >27 %) is suggested to deposit Li metal on the fully lithiated NE, a process known as ‘Li plating’.

Figure 4b and c display the NE potential for DOPL of 0 % and 50 %, during the initial cycles. Within initial 15 cycles, the potential of the NE with 50 % DOPL reaches values below 0 V *vs*. Li|Li^+^, indicating Li metal deposition, while the potential of pristine NE (0 %DOPL) remains above 0 V *vs*. Li|Li^+^, as expected. Interestingly, this Li plating region within these 15 cycles for 50 % DOPL correlates with the initial capacity rise during charge/discharge cycling in Figure 4a.

*Ex situ* ^7^Li nuclear magnetic resonance (NMR) spectra of NE and separator from NCM622 || SiNW/Gr‐A full‐cells are shown in Figure 4d. Both, the NE and separator are absent of Li metal peak, which should be observed at ~250 ppm.^[39]^ The good kinetics of thin SiNW can be speculated to realize over‐lithiated Si and prevent relevant amount of Li plating. Indeed, such over‐lithiated Li_15*+δ*_Si_4_ phase is reported and observed *via in situ* NMR.^[40]^ Nevertheless, the de‐lithiation kinetics of this phase are poor, which lead to only a slow but steadily capacity increase in Figure 4a for high DOPLs.

### Investigation of ‘Transfer‐lithiation’ between Graphite and Silicon in SiNW/Gr

**Figure 5:**
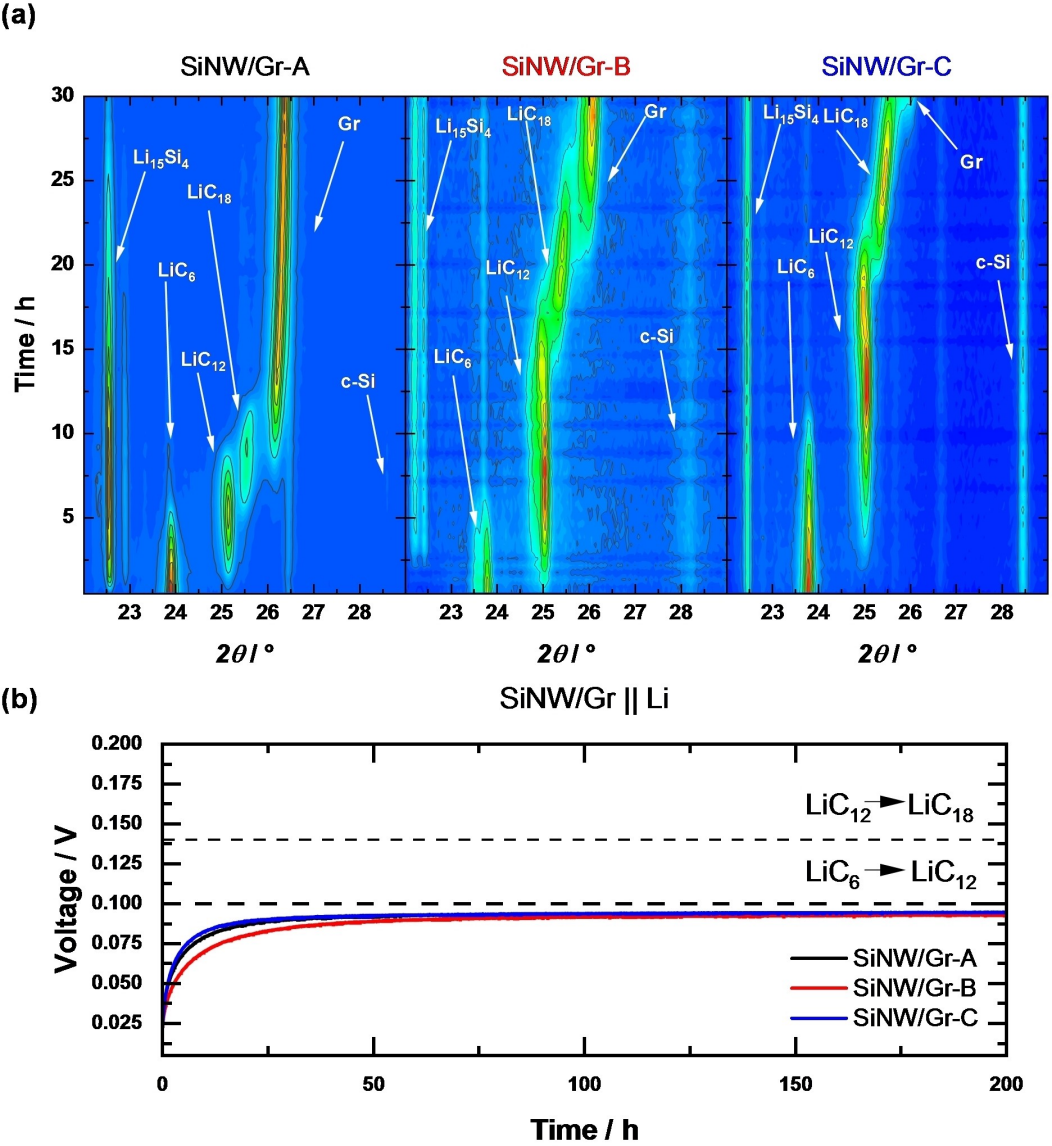
**a)** *In situ* XRD measurements of fully lithiated SiNW/Gr NEs over time, **b)** corresponding voltage *vs*. time profiles indicating absence of self‐discharge and the presence of the LiC_6_ phase.

The *in situ* XRD pattern of various NEs over time are shown in Figure 5a. All SiNW/Gr materials are fully lithiated as indicated by the *c*‐Li_15_Si_4_ and LiC_6_ characteristic reflections (at 2*θ* values of 22.5° and 23.8°, respectively) at the measurement begin. Afterwards, reflections emerge for LiC_12_ and LiC_18_ at 2*θ* values of 25° and 25.5°, while LiC_6_ diminishes. A possible self‐discharge can be excluded as shown by the open‐circuit voltage evolutions of SiNW/Gr || Li metal cells (Figure 5b). Interestingly, voltage <100 mV points to presence of LiC_6_ phase within the shown 200 h, while XRD suggests LiC_6_ absence after ≈10 h for all NEs. Here, it should be noted that *in situ* XRD requires a thicker Be‐based disc, which affects the penetration depth of X‐rays, rendering this method only surface‐sensitive, while OCV reflects entire cell, *i. e*., including bulk.

**Figure 6:**
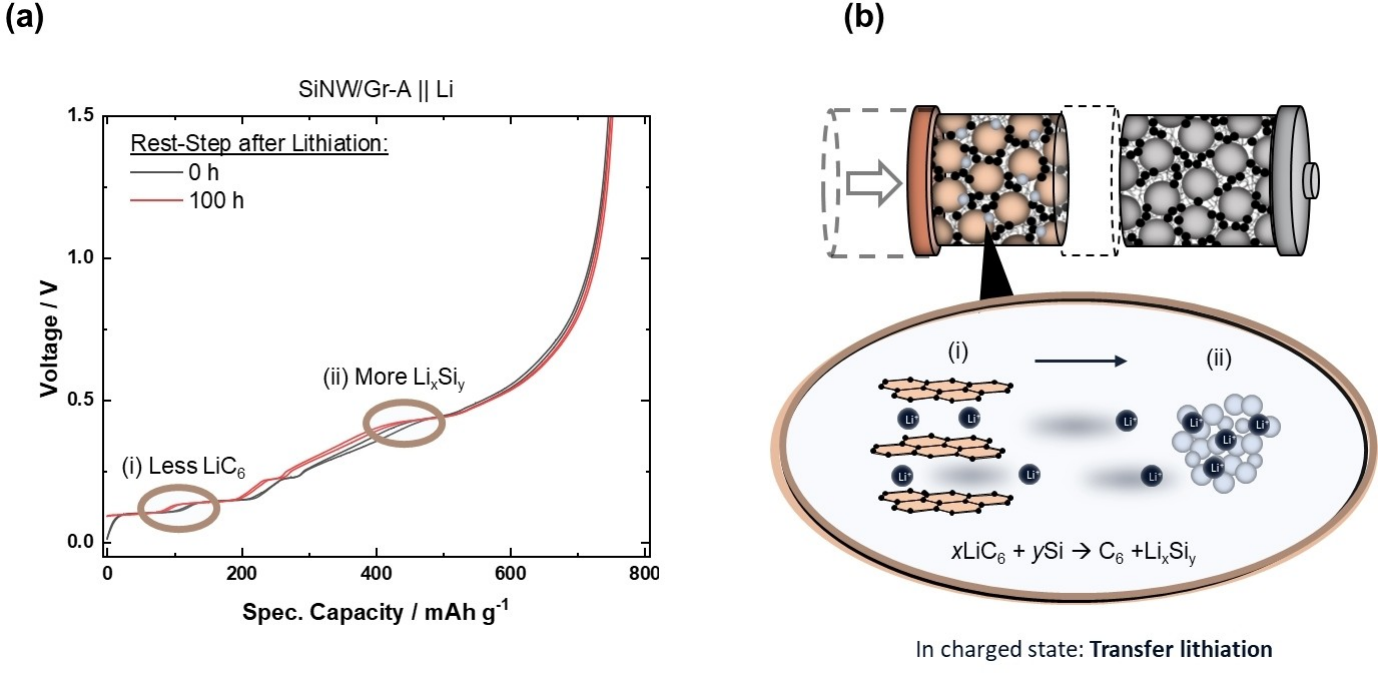
(a) Voltage *vs*. capacity profiles of SiNW/Gr‐A || Li metal cells directly de‐lithiated (black) and de‐lithiated after 100 h (red). The evolution of the discharge indicates less capacity for LiC_6_ (i) de‐lithiation after a resting period of 100 h and higher capacity for Si de‐lithiation (ii), *i. e*., transfer lithiation between lithiated Gr and Si, (b) as schematically shown.

Absence of self‐discharge is additionally confirmed by comparing specific discharge capacity/profile without and with 100 h resting at charged state (Figure 6), where the total de‐lithiation amounts are equal, rendering self‐discharge unlikely. Interestingly, the capacity contributions at respective de‐lithiation plateaus differ between the NEs, *i. e*., it lowers for (i) LiC_6_ de‐intercalation and enhances for (ii) Si de‐lithiation at 450 mV for NEs, finally pointing to an *intra*‐electrode Li/capacity shift from lithiated Gr to lithiated Si, as it is typical for a local element configuration a process, we propose to name “transfer‐lithiation”.

**Figure 7:**
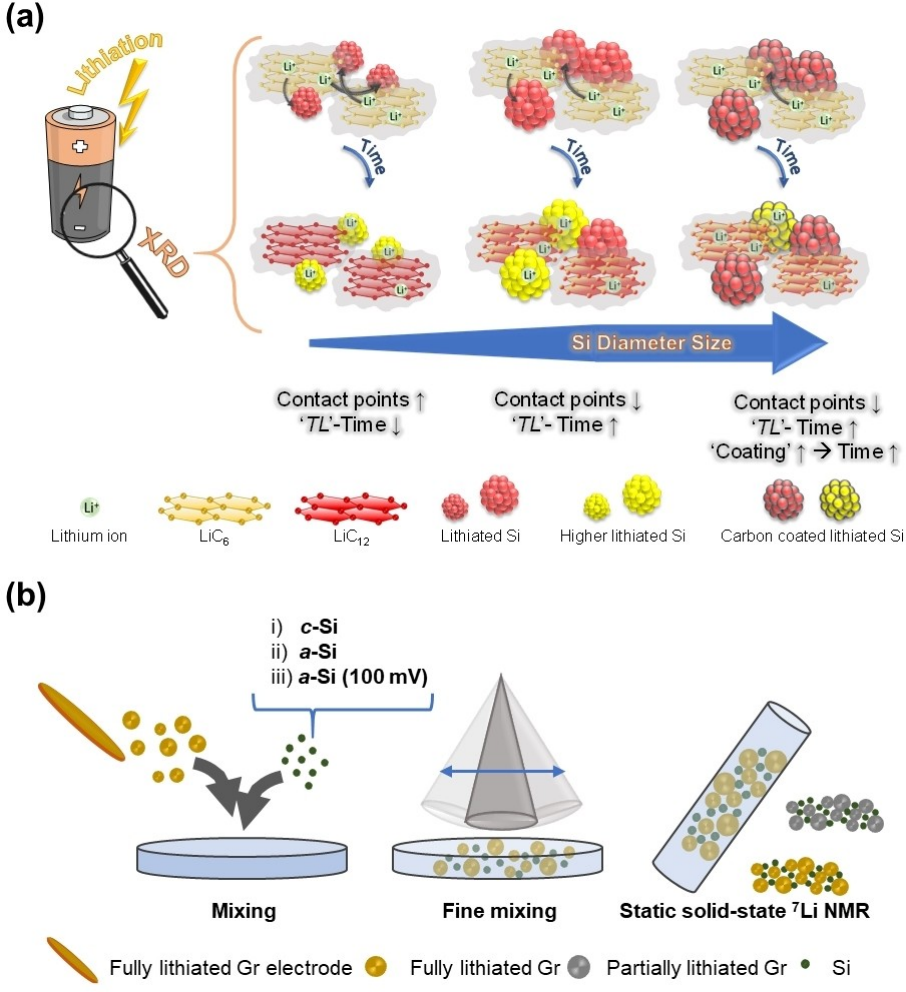
**a)** Schematic illustration of the proposed ‘transfer‐lithiation’ of Gr with different SiNWs. It is proposed that during the resting period, fully lithiated Gr (LiC_6_) transfers Li and further lithiates Si as a result of a local element configuration. Based on the lower diameter and more contact points between small size Si and Gr, the process is faster for smaller diameter of Si wires. **b)** Powder from fully lithiated Gr electrodes is mixed with i) c‐Si, ii) a‐Si (already cycled for one cycle, *i. e*., “aged”) and iii) a‐Si lithiated to 100 mV. After mixing static solid‐state ^7^Li NMR experiments are conducted.

The faster depletion of LiC_6_ with SiNW/Gr‐A compared to other NEs, seen *via in situ* XRD (Figure 5), points to a faster transfer‐lithiation, which can be related with improved kinetics due to thinner SiNW (improved transport kinetics within Si as well as possibly a better *inter* particle‐wire contact between Si and Gr, *etc*.) The SiNW/Gr‐B and SiNW/Gr‐C are similar in ‐diameter size though, but the latter is more impeded, likely due to carbon coating, as schematically summarized in Figure 7a.

**Figure 8:**
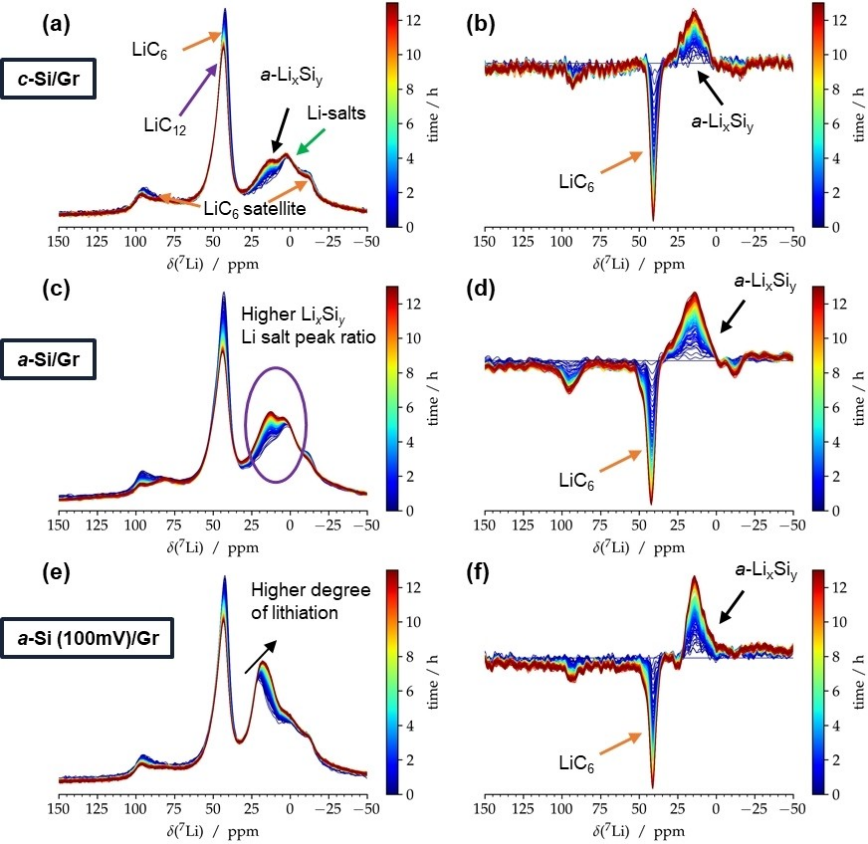
Static solid‐state ^7^Li NMR measurements of fully lithiated graphite mixed with **a)** *c*‐Si, **c)** *a*‐Si, **e)** Si powder lithiated at 100 mV at *B*_0_=11.74 T and T=328 K. **b**,**d**,**f)** show the corresponding temporal changes of peak areas as the difference of spectra with the respective ^7^Li NMR spectrum acquired first (at t=0 h). Hence, positive intensity shows an increasing signal, negative intensity a decreasing signal, respectively. Note that the ^7^Li resonance of LiC_6_ also shows a characteristic satellite transition powder pattern due to non‐negligible quadrupolar coupling.

For reasons of elucidating this phenomenon, lithiated Gr, previously charged in Gr||Li cells is detached and mixed with either pristine *c*‐Si powder, cycled *a*‐Si powder or lithiated (at 100 mV) Si powder and investigated *via* static solid‐state ^7^Li NMR according to the scheme in Figure 7b). Figure 8a displays the ^7^Li NMR spectra obtained by mixing *c*‐Si powder with lithiated graphite over time. A clear decrease of the ^7^Li peak representing LiC_6_ (~43 ppm) and the corresponding satellite peaks (~95 ppm and −10 ppm) is observed while a new ^7^Li peak in the range from 15–12 ppm emerges, which corresponds to lithiated Si (Li_*x*_Si_*y*_); note that this peak might also overlap with the signal from LiC_18_. For clarity reasons, Figure 8b illustrates the increase (positive values) and decrease (negative values) of the respective ^7^Li NMR signals.

Transfer‐lithiation with amorphous, *i. e*., cycled Si (Figure 8c and d) seems to be faster as seen by enhanced peak differences for a given time. Moreover, the increase in peak ratio of *a*–Li_*x*_Si_*y*_ to residual Li‐salts such as LiPF_6_ or SEI compounds (~0 ppm) is higher compared to *c*‐Si (Figure 8a) and hints to higher amount/kinetics of transfer‐lithiation, likely due to a lowered activation barrier. This scenario is more practical‐near compared to *c*‐Si in Si/Gr composite‐based NEs, as cycled Si is predominantly present as *a*‐Si.

To further mimic application scenarios, partially lithiated Si powder at 100 mV is paired with LiC_6_, which also suggests transfer‐lithiation as seen by similar shifts in respective phases (cf. Figure 8e and f). Interestingly, the ^7^Li peak for *a*–Li_*x*_Si_*y*_, being present from the beginning as expected, shifts towards lower *δ*(^7^Li) values indicating higher lithiated Li_*x*_Si_*y*_ phases,^[41]^ and can be concluded to be faster compared to *c*‐Si and *a*‐Si.

**Figure 9:**
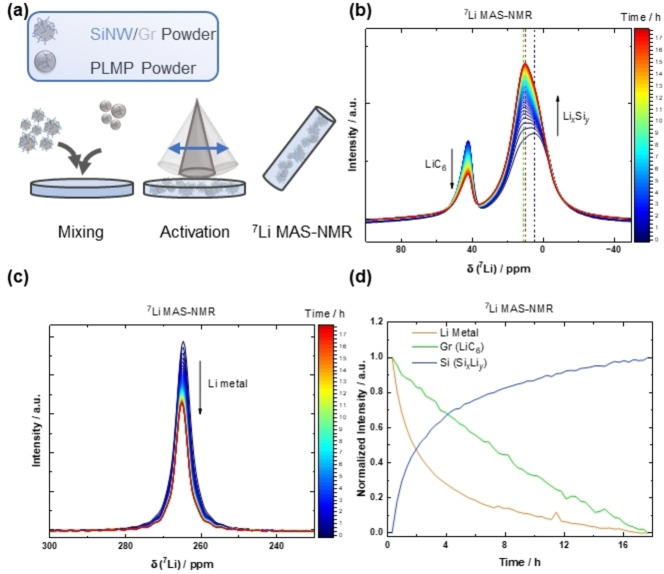
**a)** Schematic illustration of the preparation of SiNW/Gr‐A composite with PLMP for ^7^Li MAS NMR measurements. **b)** Evolution of the lithiated Gr and lithiated Si signals over time, **c)** evolution of Li metal from PLMP over time and **d)** normalized integrated peak areas for each measurement pointing to transfer lithiation.

To further validate the process of ‘transfer–lithiation’, SiNW/Gr‐A‐based NE is pre‐lithiated by direct contact with passivated lithium metal powder (PLMP) and afterwards investigated *via* ^7^Li magic angle spinning (MAS) NMR spectroscopy (Figure 9a). At the beginning of the measurement (blue, Figure 9b), *c*‐Li_15_Si_4_ is indicated at ≈6 ppm and *a*‐Li_13_Si_4_ *via* second shoulder peak at ≈11 ppm.^[41]^ The latter peak increases in intensity while the *c*‐Li_15_Si_4_ peak decreases over time. The presence of *c*‐Li_15_Si_4_ can be related to local lithiation hotspots from pressed PLMP resulting in highly‐ and over‐lithiated Li_*x*_Si_*y*_ phases.[^40^, ^42^] As seen by the steadily shift to higher *δ*(^7^Li) values, Li^+^ is redistributed and decrease the over‐lithiated Li_15+δ_Si_4_ phase (static NMR, Figure S6) more effectively compared to Li^+^ contribution from PLMP (Figure 9c), which would shift to opposite, *i. e*., higher *δ*(^7^Li) values.

The plot of integrated peak areas (Figure 9d) indicates correlation between increase of lithiated Si and decrease of lithiated Gr and Li. Within the first four to six hours the curves of decreasing Li metal and increasing lithiated Si are nearly mirror‐inverted. Afterwards, there is also a slight linear increase for lithiated Si correlating to a linear decrease of the lithiated Gr‐peak area, likely because of Gr de‐lithiation (transfer‐lithiation) as seen by shrinking peak area over time (Figure 9a).

Once Si is fully lithiated, the ^7^Li peak intensity of Gr still decreases linearly, while Li_*x*_Si_*y*_ proceed to shift towards lower *δ*(^7^Li) values, *i. e*., towards higher degree of lithiation. ‘Transfer–lithiation’ therefore also occurs during NE‐lithiation process seen in the example of direct contact pre‐lithiation with PLMP.

### Self‐discharge vs. Transfer‐lithiation

In literature it is known, that formation of *c*–Li_15_Si_4_ phase enhances self‐discharge due to continuous SEI growth, which is accompanied by phase transition from c‐Li_15_Si_4_ to a‐Li_*x*_Si_*y*_.^[43]^ However, in this study, a PET‐containing *Separion P20* separator is used in combination with electrolyte w/o additives.^[43]^ This indeed can lead to self‐discharge *via* redox shuttle of dimethyl terephthalate, when PET is present.[^44^, ^45^] A recent study from *Arca et al*. shows PF_6_ salt decomposition leading to instable SEI on Si surfaces, de‐lithiating Si and therefore facilitating the ‘transfer–lithiation’ from Gr to Si.^[46]^ Nevertheless, in this study no change in capacity is observed for SiNW/Gr‐A between 0 and 100 hours (Figure 6), disproving relevance of self‐discharge at these conditions.

## Conclusions

A decrease in diameter size of silicon nanowires (SiNWs) on graphite within a composite anode increase specific discharge capacities and cycle life in Li half cells, but is accompanied by enhanced active lithium loss (ALL) due to their higher specific surface area (SSA). In full cell application, this material is therefore pre‐lithiated to compensate ALL.

**Figure 10:**
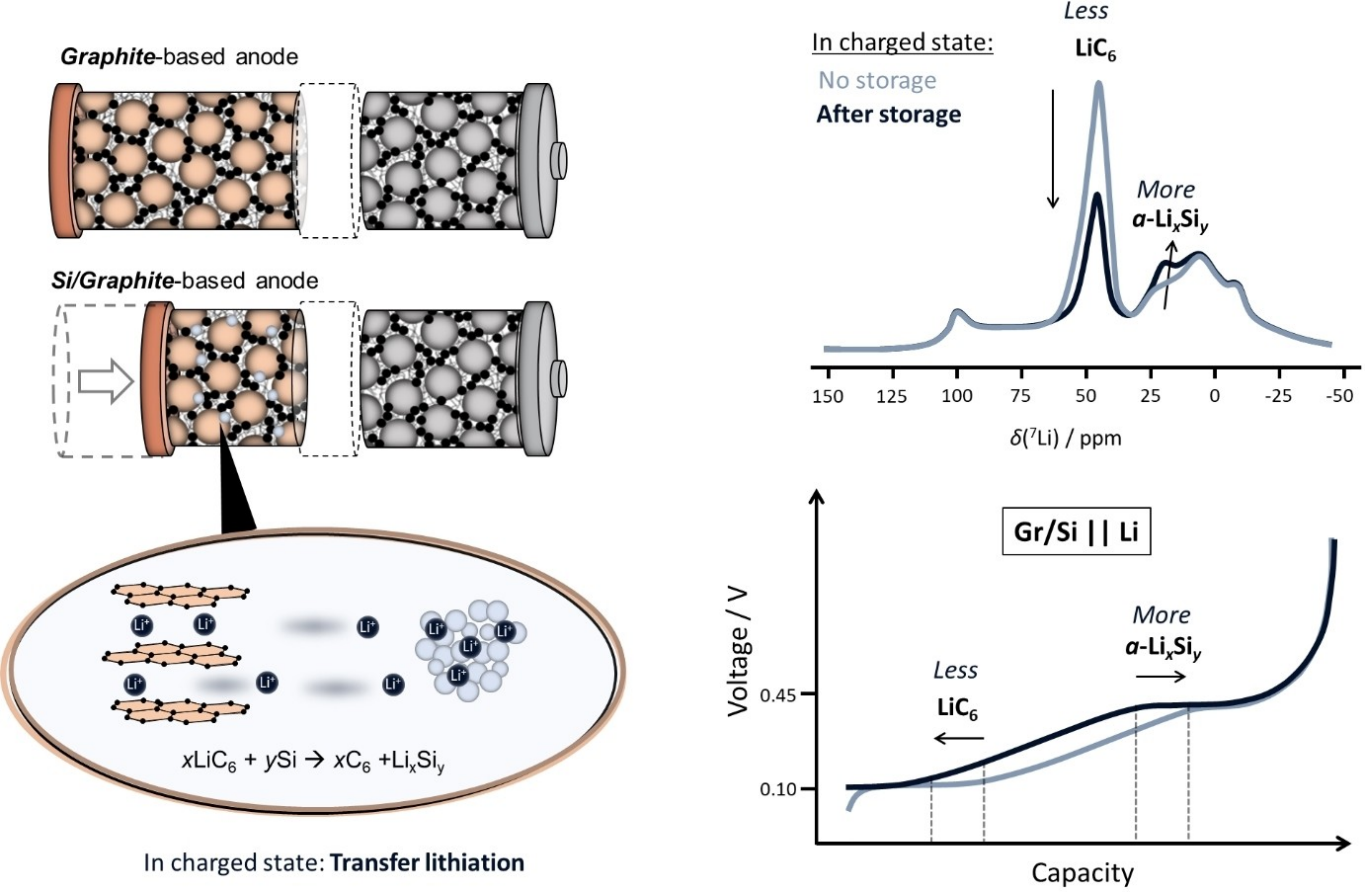
Incorporating Si in a graphite‐based anode can enhance the energy density, though Li^+^ re‐destribution between graphite and Si in the charged (or pre‐lithiated) state should not be disregarded, as overlithiation of Si can occur. This transfer‐lithiation from lithiated graphite (LiC_6_) to Si (a typical local element) is proven, among others, *via* NMR and galvanostatically after pre‐lithiation and/or storage experiments.

Interestingly, despite potentials below 0 V *vs*.Li|Li^+^ no evidence for metallic, *i. e*., plated, Li is observed *via* nuclear magnetic resonance spectroscopy (NMR), and suggests a local element between Gr and Si, here named “transfer‐lithiation”. Simple storage experiments indeed indicate transfer lithiation by shifts in capacity of respective potential plateaus, *i. e*., lowered capacity for LiC_6_ (at 0.1 V) and enhanced capacity for α‐Li_*x*_Si_*y*_ (at 0.45 V) as well as respective shifts in NMR (Figure 10), *i. e*., decrease of peak at 43 ppm and increase at 12 ppm, as shown in Figure 10. Here, self‐discharge during storage is excluded as seen by similar specific discharge capacities after storage.

Validation experiments *via* ^7^Li nuclear magnetic resonance (NMR) spectroscopy by varying Si morphologies during prelithiation with passivated lithium metal powder (PLMP) also reveal structure‐related velocity of ‘transfer‐lithiation’, which is faster for amorphous (*a*)‐Si compared to crystalline Si.

## Conflict of Interests

The authors declare no conflict of interest.

1

## Supporting information

As a service to our authors and readers, this journal provides supporting information supplied by the authors. Such materials are peer reviewed and may be re‐organized for online delivery, but are not copy‐edited or typeset. Technical support issues arising from supporting information (other than missing files) should be addressed to the authors.

Supporting Information

## Data Availability

The data that support the findings of this study are available from the corresponding author upon reasonable request.
