# Supplementary material for: Elucidating ‘Transfer‐Lithiation’ from Graphite to Si within Composite Anodes during Pre‐Lithiation and Regular Charging
Source: ChemSusChem. 2024 Dec 19;18(7):e202401290. doi: 10.1002/cssc.202401290 (PMC11960597; doi:10.1002/cssc.202401290)
Supplement: Supplementary file 1 — Supporting Information [file CSSC-18-e202401290-s001.pdf]

ChemSusChem

Supporting Information

Elucidating ‘Transfer-Lithiation’ from Graphite to Si within Composite Anodes during Pre-Lithiation and Regular Charging

Lars Frankenstein, Pascal Jan Glomb, Marvin Mohrhardt, Steffen Böckmann, Leon Focks, Aurora Gomez-Martin, Tobias Placke, Michael Ryan Hansen, Martin Winter, and Johannes Kasnatscheew*

Supplementary Information

Elucidating 'Transfer-Lithiation' from Graphite to Si within Composite Anodes during Pre-Lithiation and Regular Charging

Lars Frankenstein[a], Pascal Jan Glomb[b], Marvin Mohrhardt[b], Steffen Böckmann[c], Leon Focks[a], Aurora Gomez-Martin[a], Tobias Placke[a], Michael Ryan Hansen[c], Martin Winter[a,b,*], Johannes Kasnatscheew[a,*]

Affiliations:

[a] University of Münster, MEET Battery Research Center, Institute of Physical Chemistry, Corrensstraße 46, 48149 Münster, Germany

[b] Helmholtz Institute Münster, IEK-12, Forschungszentrum Jülich GmbH, Corrensstraße 46, 48149 Münster, Germany

[c] University of Münster, Institute of Physical Chemistry, Corrensstraße 28/30, 48149 Münster, Germany

[*] Corresponding authors: j.kasnatscheew@uni-muenster.de; martin.winter@uni-muenster.de

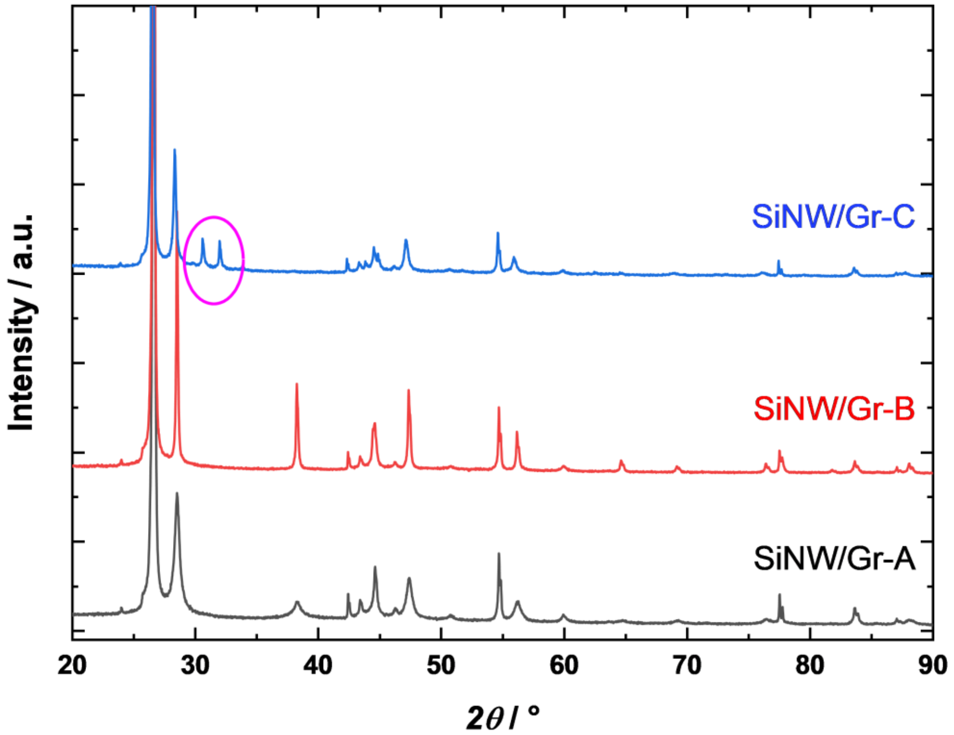

Figure S 1: *X-ray diffraction patterns of the three SiNW/Gr-based powders. The presence of Sn in SiNW/Gr-C is confirmed by additional reflections marked by magenta-colored circle.*

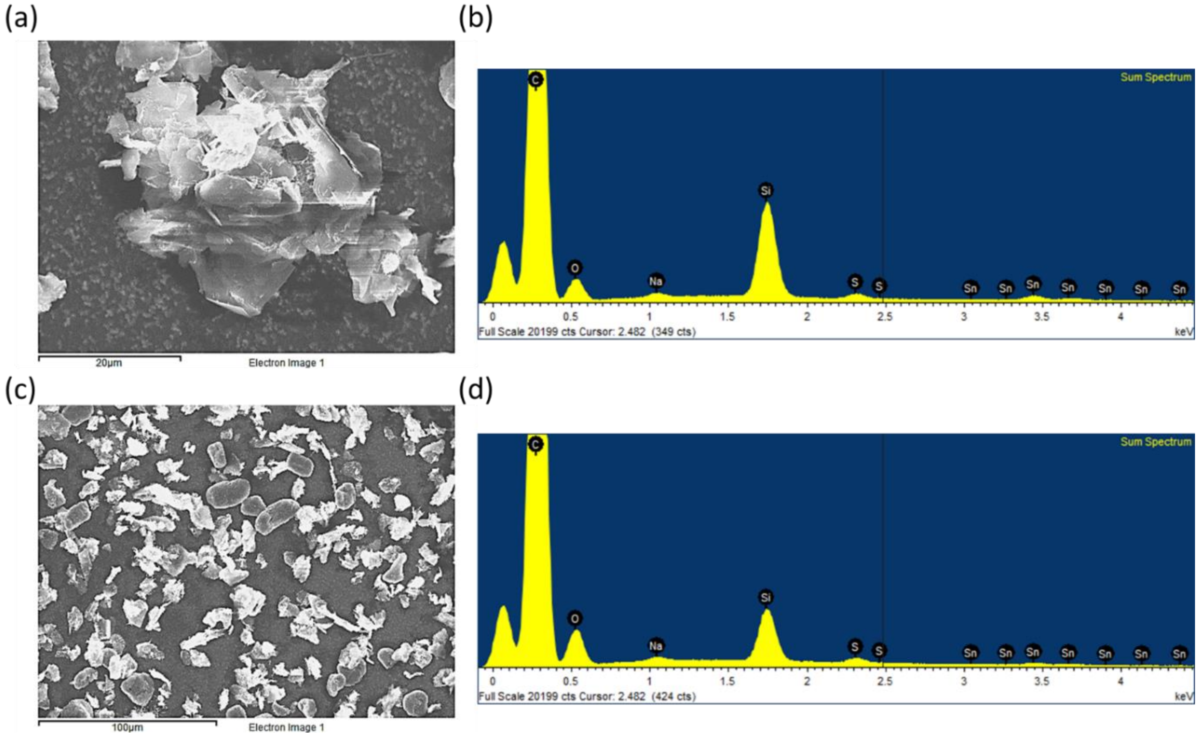

Figure S 2: ***a)*** *SEM image of a particle cluster of SiNW/Gr-C at high acceleration voltages to measure EDX.* ***b)*** *EDX element spectrum of the particle cluster.* ***c)*** *SEM image of many particles of SiNW/Gr-C at high acceleration voltages to get an averaged element distribution.* ***d)*** *EDX element spectrum of many particles.*

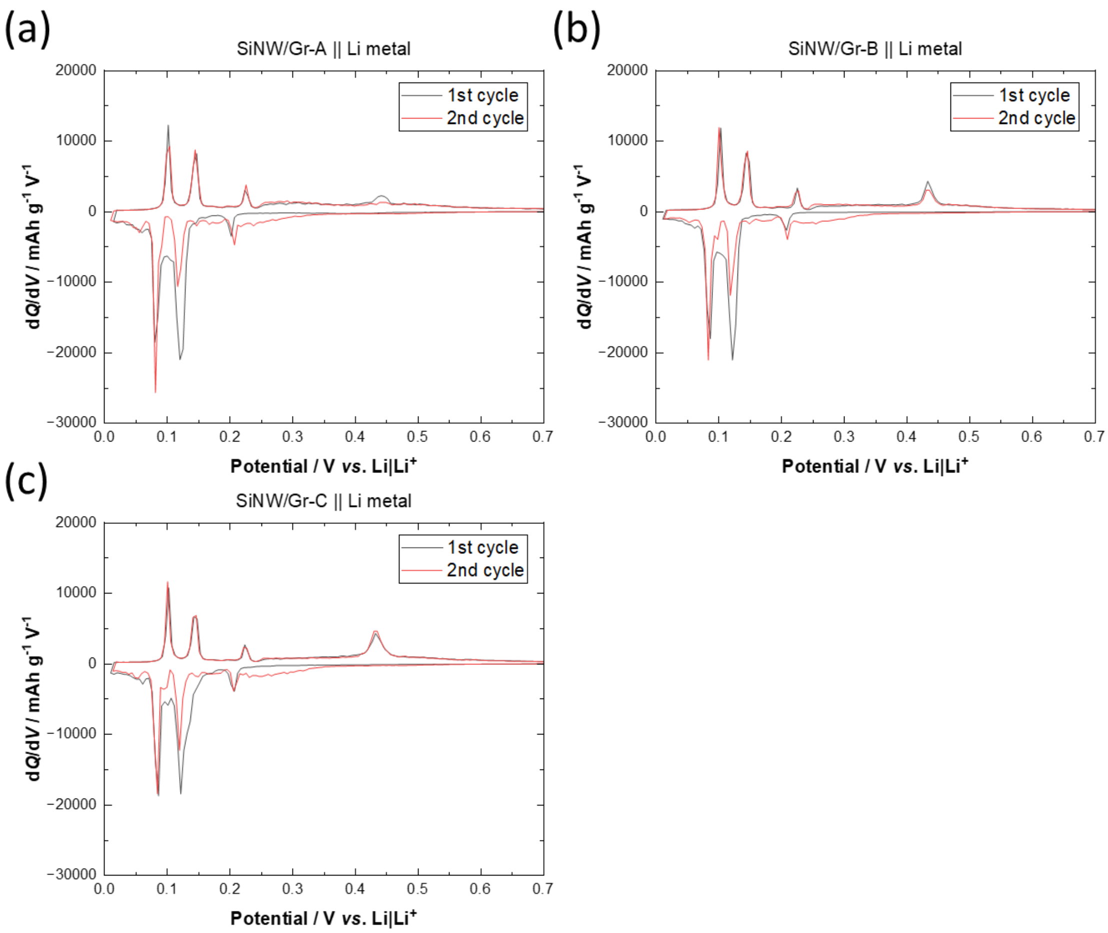

***Figure S 3**: Differential capacity vs. potential profiles of first cycle (black) and second cycle (red) for **a)** SiNW/Gr-A, **b)** SiNW/Gr-B and **c)** SiNW/Gr-C. All dQ/dV profiles are recorded in three-electrode configuration with Li metal as counter and reference electrodes.*

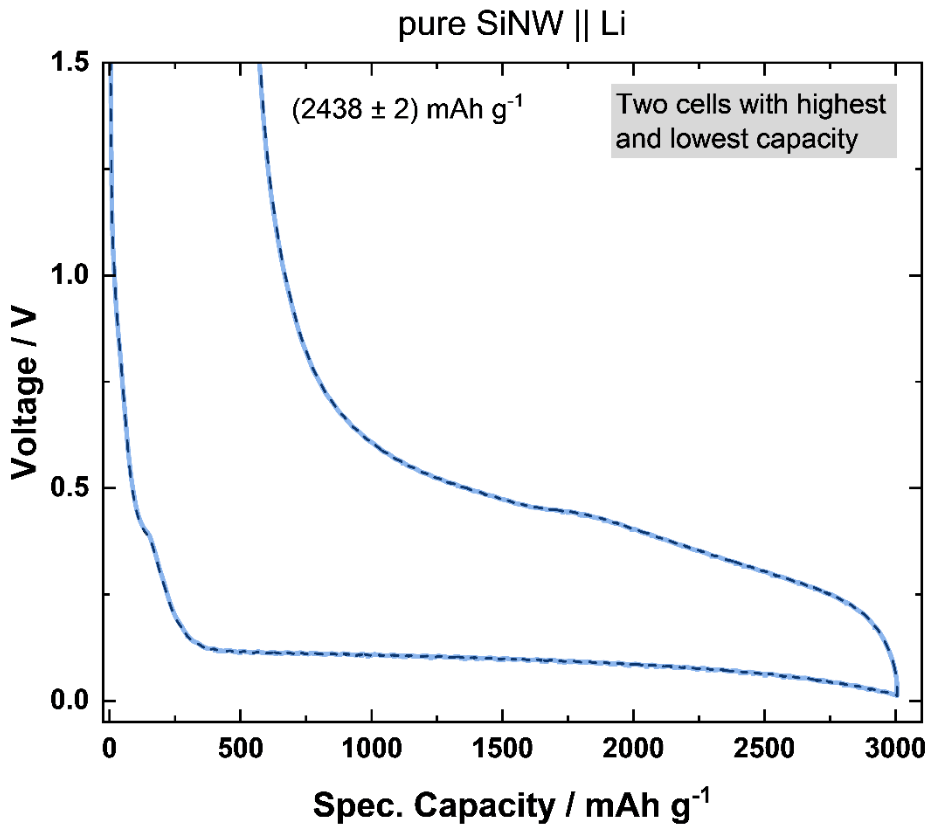

Figure S 4: *Voltage profiles of pure SiNWs indicating practical specific delithiation capacity of 2438 ± 2 mAh g^{-1}. The voltage profile of pure SiNW is measured in two-electrode configuration using low current of 0.05C (1C = 3000 mA g^{-1}).*

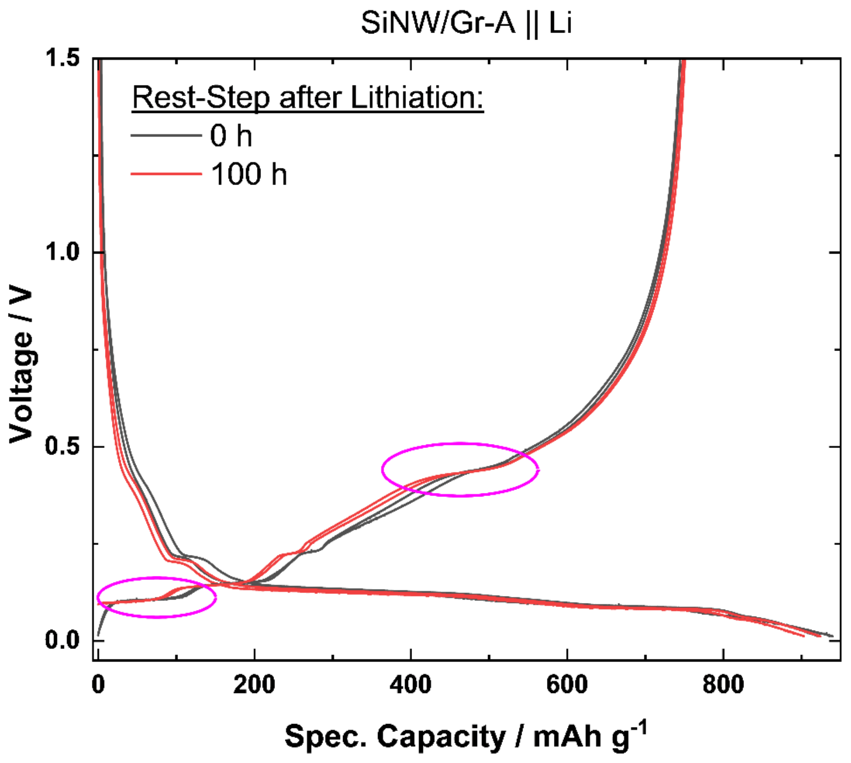

Figure S 5: *Voltage vs. capacity profiles of SiNW/Gr-A without CV-step during lithiation at 0.01 V followed by direct delithiation (black) and delithiation after 200 h in rest step (red). The magenta-colored circle indicates an increase in the plateau representing the c-$Li_{15}Si_4$ phase explained by the observed transfer-lithiation. The voltage profile is measured in two-electrode configuration using low current of 0.05C (1C = 800 mA g^{-1}).*

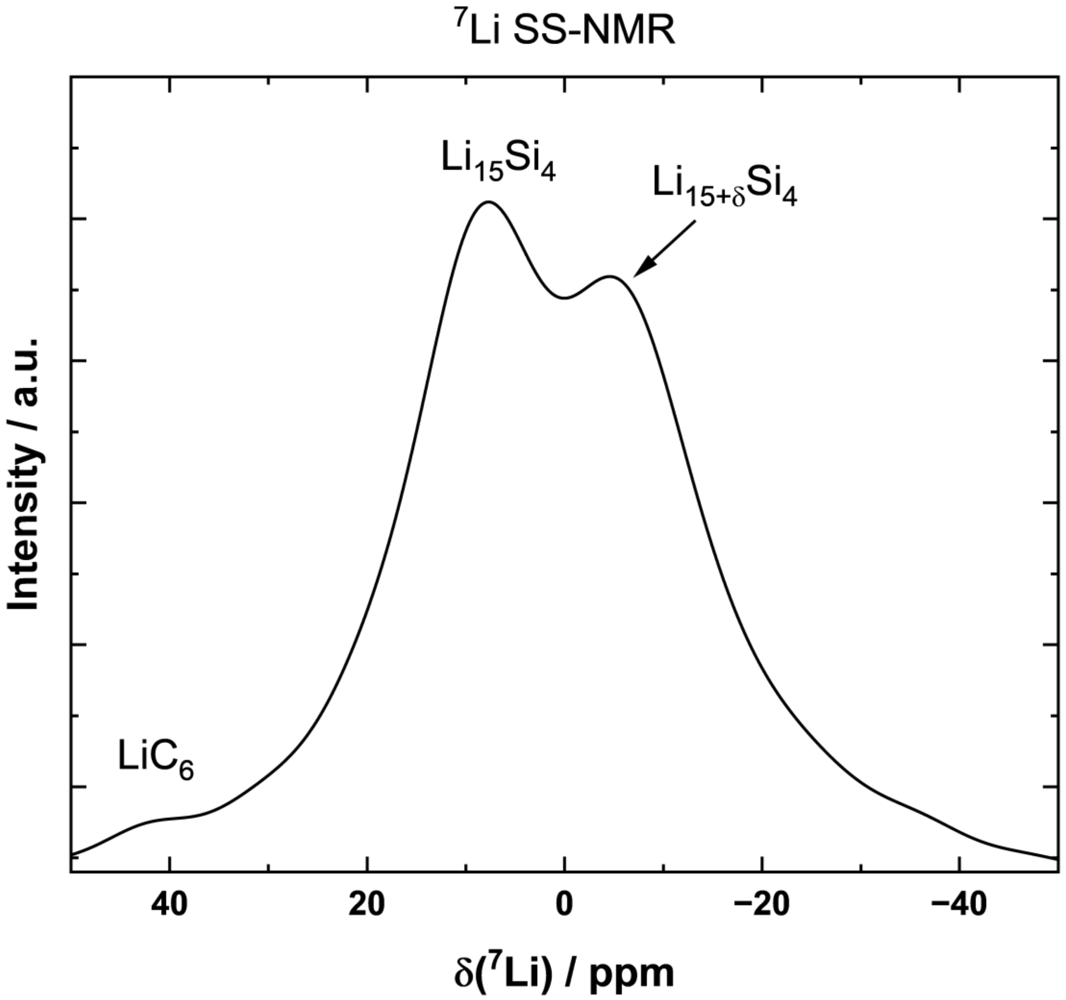

Figure S 6: *^{7}Li SS-NMR measurements of SiNW/Gr-A mixed with PLMP to identify over-lithiated $Li_{15+\delta}Si_4$ phase prior to ^{7}Li MAS-NMR measurements in* ***Figure 9****.*
